# Dual Kinase Targeting in Leukemia

**DOI:** 10.3390/cancers13010119

**Published:** 2021-01-01

**Authors:** Luca Mologni, Giovanni Marzaro, Sara Redaelli, Alfonso Zambon

**Affiliations:** 1 Department of Medicine and Surgery, University of Milano-Bicocca, 20900 Monza, Italy; luca.mologni@unimib.it (L.M.); sara.redaelli@unimib.it (S.R.); 2 Department of Pharmaceutical and Pharmacological Sciences, University of Padova, via Marzolo 5, I-35131 Padova, Italy; giovanni.marzaro@unipd.it; 3 Department of Chemistry and Geological Sciences, University of Modena and Reggio Emilia, 41125 Modena, Italy

**Keywords:** multi-kinase inhibitors, AML, CML, lymphoma, PROTAC

## Abstract

**Simple Summary:**

A new option to treat cancer is based on the use of so-called multi-targeting drugs. This strategy can replace the standard treatment based on the co-administration of several drugs. An increased and uncontrolled activity of kinases (enzymes devoted to the regulation of several cell pathways) is often seen in hematological malignancies. The development of multi-kinase inhibitors is having a great impact on the treatment of this kind of cancer. Here, we review the most recent findings on this novel class of drugs.

**Abstract:**

Pharmacological cancer therapy is often based on the concurrent inhibition of different survival pathways to improve treatment outcomes and to reduce the risk of relapses. While this strategy is traditionally pursued only through the co-administration of several drugs, the recent development of multi-targeting drugs (i.e., compounds intrinsically able to simultaneously target several macromolecules involved in cancer onset) has had a dramatic impact on cancer treatment. This review focuses on the most recent developments in dual-kinase inhibitors used in acute myeloid leukemia (AML), chronic myelogenous leukemia (CML), and lymphoid tumors, giving details on preclinical studies as well as ongoing clinical trials. A brief overview of dual-targeting inhibitors (kinase/histone deacetylase (HDAC) and kinase/tubulin polymerization inhibitors) applied to leukemia is also given. Finally, the very recently developed Proteolysis Targeting Chimeras (PROTAC)-based kinase inhibitors are presented.

## 1. Introduction

**Figure 1:**
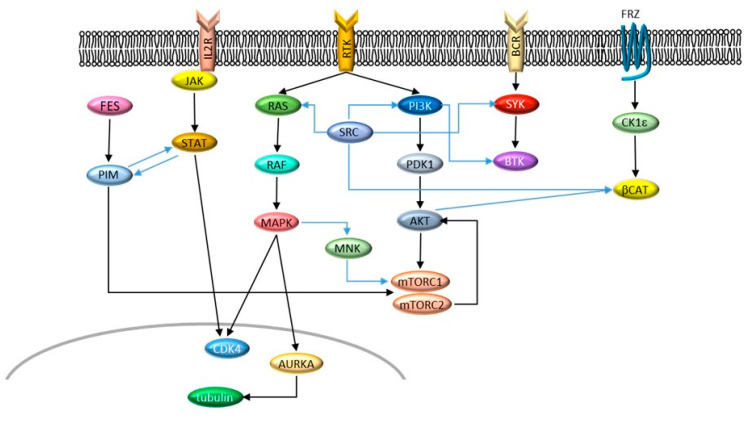
Signaling networks described in this review. Cross-talks are indicated by blue arrows.

One of the most spectacular breakthroughs in cancer therapy in the past 20 years is undoubtedly represented by the introduction of imatinib mesylate for the treatment of Philadelphia-positive (Ph+) leukemia [1,2]. The success of imatinib instilled the idea that most tumors could be treated by a single, targeted drug that inhibits the main driver of transformation. However, the initial enthusiasm subsided when it became clear that, on the contrary, most tumors harbor at least two or more driver lesions, all of which need to be targeted to achieve disease control [3]. Even when one major driver can be identified, cells can often switch to alternative signaling to resist therapy [4] (Figure 1). Therefore, dual targeting is being intensively investigated to improve treatment outcomes [5,6,7]. The rationale behind the better anticancer activity of combined inhibition is based on (i) complete and simultaneous suppression of cooperating oncogenic signals and (ii) concurrent block of bypass signaling tracks that may mediate drug resistance. Obviously, inhibiting only one of two cooperating oncogenic signals will produce a partial effect. Moreover, a cancer cell is less likely to develop resistance to two drugs than to one (probability of dual resistance is the product of the probabilities of single resistance). In this scenario, dually targeted molecules represent a further step: They achieve combined targeting without the unwanted combination of side effects that can be observed in traditional dual targeting by two different molecules. From a medicinal chemistry point of view, “dual” may have different meanings: researchers can develop a dual inhibitor by intentionally aiming at two specific targets or simply by exploiting off-target activity of a compound primarily targeted to another kinase. As drugs are most often not mono-specific, off-targets can be detrimental, beneficial, or irrelevant to the disease under study. Thus, in general, “dual” indicates a drug that targets two relevant kinases at comparable potency, showing some selectivity over other unwanted targets. This review provides an overview of the development of dual inhibitors of kinase targets for the treatment of leukemia.

### 1.1. Dual Kinase Targeting in AML

Acute myeloid leukemia (AML) is a cancer defined by the infiltration of bone marrow, blood, and other tissues by proliferative, clonal, abnormally differentiated and poorly differentiated cells of the hematopoietic system [8]. AML is characterized by cytogenetic and molecular heterogeneity; genetic abnormalities include amplifications, deletions, rearrangements, and point mutations [8,9]. The last WHO classification has added RUNX1 mutations and BCR-ABL1 rearrangements to the previous main molecular markers (NPM1, CEBPA, and FLT3) [10]. Most AML patients respond well to chemotherapy, but subsequent relapse is common, and the prognosis is still poor, with approximately 30% of adult patients under 60 years of age achieving 5-year survival and a median survival of 5 to 10 months for older patients [11,12,13].

New, more targeted therapeutic approaches have been attempted to meet this clinical need, such as the use of fms-like tyrosine kinase 3 (FLT3) inhibitor midostaurin [14,15], isocitrate dehydrogenase-2 (IDH2) inhibitor enasidenib [16], and calicheamicin conjugated anti-CD33-antibody gemtuzumab [17]. As commonly observed, the major limitation of targeting these individual oncogenes is the development of resistance; hence, long-term remissions in AML still require the combined use of cytotoxic chemotherapy and/or targeted agents along with stem cell transplant, with ensuing systemic toxicity [11]. The dual inhibition of protein kinases in AML has been extensively explored to achieve response to relapsing and resistant tumors avoiding toxic side effects, mainly involving co-inhibition of the phosphatidylinositol 3-kinase(PI3K)/protein kinase B (AKT)/mechanistic target of rapamycin (mTOR) (PI3K/AKT/mTOR) pathway and FLT3 kinase. A summary of the dual kinase targeting molecules proposed for the treatment of AML is presented in Table 1 at the end of this section.

In hematopoietic cells, the PI3K/AKT/mTOR pathway is an important regulator of cell growth, proliferation, and survival; the amplification and deregulation of this pathway confer resistance to apoptosis, growth inhibition, and differentiation on leukemia cells [18]. Constitutive activation of the PI3K/AKT/mTOR signal is common in AML patients and linked to reduced survival, making the targeting of its components a prime strategy for the treatment of AML [11,19]. The PI3K family of lipid kinases comprises several regulatory and catalytic subunits encoded by different genes [20]. PI3K is the first enzyme along the PI3K/AKT/mTOR pathway to be activated by receptor tyrosine kinases (RTKs) or through an adaptor molecule; the main group of PI3Ks involved in neoplastic diseases is class I PI3K [18]. The mTOR protein is involved in two major complexes: the mTOR complex 1 (mTORC1) and the mTOR complex 2 (mTORC2). The mTORC1 controls major regulators of cellular growth and proliferation like the cyclin-dependent kinase inhibitor p27kip1, the retinoblastoma protein (Rb), cyclin D1, c-myc, and STAT3, while the mTORC2 complex has a role in regulating AKT and in resistance to apoptosis [21]. The mTOR pathway helps regulate several key cellular processes, including cell growth, proliferation and cycling, and protein synthesis. In AML, dysregulation of these processes aids leukemia cells in enhancing growth and proliferation and resisting apoptosis; increased mTOR activity thus plays a role in AML relapse and initiation [21,22]. The first mTOR inhibitor discovered was rapamycin; rapamycin and its analogs (rapalogs) bind specifically to the FKBP12-rapamycin-binding (FRB) domain of mTOR, preventing the formation of the mTORC1 complex and are thus mTORC1- specific [23]. While preclinical studies on mTORC1 inhibitors have shown that rapamycin and its analogs are able to slow the growth and proliferation of AML cell lines and leukemic blasts [24], in numerous clinical studies on AML patients, they have shown a very limited efficacy when used as monotherapies [11], and even their combination with conventional chemotherapies did not yield a clear advantage against the standard of care [25,26,27].

Among the mechanisms of resistance to mTORC1 inhibitors, feedback activation of PI3K/AKT signaling upon mTORC1 inhibition is of particular therapeutic interest [28]. When only mTORC1 and not mTORC2 is inhibited, AML cells exhibit increased AKT phosphorylation, amplifying survival signals, and activating PI3K [29]. This paradoxical activation contributes to the lack of efficacy of rapamycin and rapalogs and provides a strong rationale for the concomitant inhibition of mTORC1 and mTORC2 and the dual targeting of mTOR and PI3K or AKT [11].

**Figure 2:**
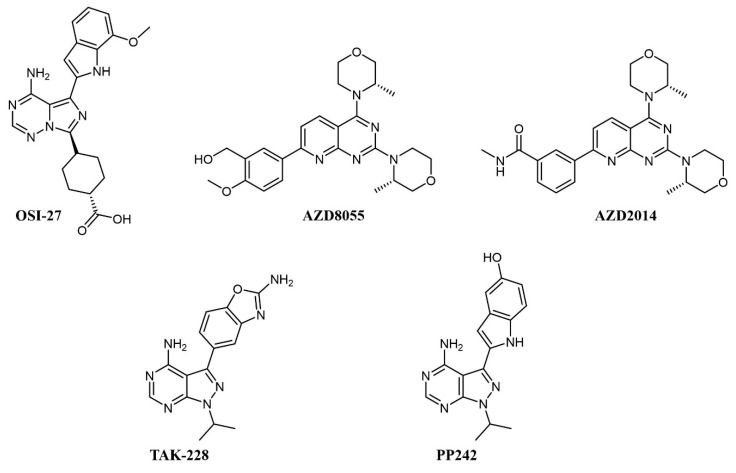
Chemical structure of dual mTOR complex 1 (mTORC1)/mTORC2 inhibitors used for acute myeloid leukemia (AML) treatment.

Simultaneous inhibition of mTORC1 and mTORC2 is achieved by ATP-competitive kinase inhibitors like OSI-027, AZD8055 and AZD2014, TAK-228, and PP242 (see Figure 2 for chemical structures), all of which abrogate paradoxical activation of AKT [30].

In preclinical studies on AML cell lines, OSI-027 showed significant inhibition, both as a single agent and in combination with standard chemotherapy, such as cytarabine [31,32]. In a first-in-man, dose-finding clinical trial on solid tumors, OSI-027 inhibited mTORC1/mTORC2 in a dose-dependent manner, but the maximum tolerated dose did not allow significant target engagement in tumor [33]. AZD8055 fully abrogates the PI3K feedback activation and shows remarkable efficacy and no notable toxicity on xenograft models [34]. In clinical trials, AZD8055 showed reasonable toxicity [35] but was later discontinued [36]. Its analog, AZD2014 (vistusertib), had superior pharmacokinetics [36] and a good tolerability profile [37]; on AML cells, it showed synergistic effects with the antibody-drug conjugate gemtuzumab ozogamicin by activation of lysosomal function [38] and with the pan-PIM inhibitor AZD1208 [39].

PP242 also shows better inhibition of mTORC1 and mTORC2 activity and suppression of feedback PI3K/AKT activation than rapamycin and achieved good efficacy with no toxicity in murine models [40]; similar to AZD2014, PP242 showed synergy with gemtuzumab ozogamicin by an analogous mechanism [41]. MLN0128 (formerly TAK-228) is a selective ATP-competitive mTORC1/2 inhibitor that induces apoptosis in cell lines with amplified AKT/mTOR signaling, including AML cell lines, without affecting healthy cells [42]. MLN0128 has entered various clinical trials, including against hematologic malignancies, such as relapsed lymphoma (NCT02727777), showing preliminary therapeutic activity [43].

**Figure 3:**
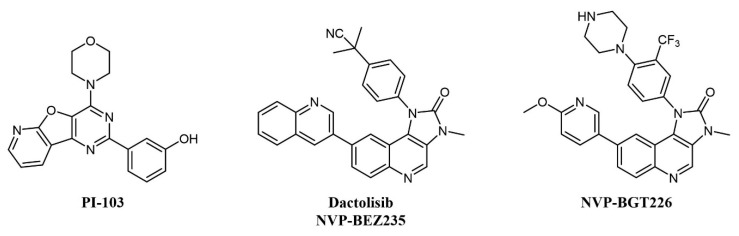
Chemical structure of dual mTOR/PI3K inhibitors used for AML treatment.

Several mTOR/PI3K dual inhibitors have been reported, many of which have been tested on AML (Figure 3) [44]; interestingly, cellular studies on primary blasts collected from AML patients evidenced that high PI3K signaling was associated with resistant samples, and dual PI3K/mTOR inactivation was proven to be cytotoxic also to leukemia-initiating cells [45].

Despite the straightforward rationale of concomitant inhibition of PI3K and mTOR, toxicity concerns have been raised over the blocking of PI3K activity, as this kinase is key in a series of important cellular processes [46]. One of the first compounds of this type to be developed was PI-103 (Figure 3), which provided proof-of-concept for the therapeutic potential of double PI3K/mTOR agents on AML cells by inhibiting leukemic proliferation, clonogenicity of leukemic progenitors, and inducing mitochondrial apoptosis [47] but did not enter clinical trials due to limited solubility and extensive metabolism [48]. Other PI3K/mTOR inhibitors assessed on AML are two imidazoquinoline analogs, dactolisib (NVP-BEZ235) and NVP-BGT226 (Figure 3). Dactolisib is endowed with pan-PI3K/mTOR specificity [49]. When tested on 21 primary AML samples and human leukemic cell lines, the compound reduced their proliferation rate, inducing a strong apoptotic response in AML cells without affecting normal CD34+ progenitor cells’ survival [50]. Interestingly, both NVP-BGT226 and dactolisib suppress AKT signaling pathways and show potent antiproliferative effects, but while NVP-BGT226 has potent pro-apoptotic effects in vitro as well as ex vivo on leukemic blasts, dactolisib causes G1/G0 arrest and prevents the induction of apoptosis [51]. Dactolisib entered a Phase I clinical trial on 22 patients with relapsed or refractory acute leukemia (NCT01756118) in 2012, with an overall response rate of 30% and a sustained molecular remission in a single patient [52].

**Figure 4:**
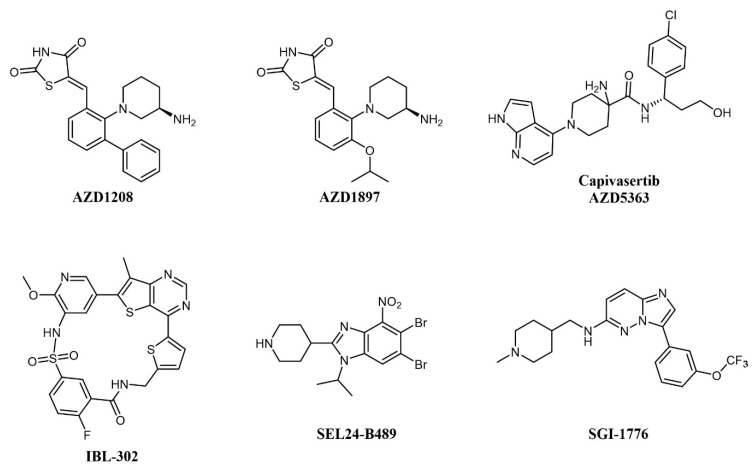
Chemical structure of multi-targeting PIM inhibitors used for AML treatment. The structure of the phosphatidylinositol 3-kinase/protein kinase B (AKT) inhibitor Capivasertib (used in combination with AZD1897) is also reported.

Concomitant dual inhibition of proviral integration site for Moloney murine leukemia virus 1 (PIM1) with other molecular targets has also been proposed as a treatment strategy in AML and evaluated in preclinical studies. PIM proteins are a family of three Ser/Thr kinases responsible for cell cycle regulation, anti-apoptotic activity, and the activation of receptor tyrosine kinases. PIM signaling has a role in defining cell fate, including senescence, cell cycle regulation, apoptosis, metabolism, invasion, and metastasis; PIM1 mRNA levels are increased in acute myeloid leukemia (AML); thus, the combination of a PIM kinase inhibitor with an mTOR inhibitor is expected to offer greater antitumor effects in AML than either inhibitor alone [53]. The combination of dual mTOR inhibitor AZD2014 and pan-PIM inhibitor AZD1208 (Figure 4) effectively reduces protein synthesis by simultaneous inhibition of the mTORC1/2 pathway and induces apoptosis in AML cells [39]. In a similar study, the PIM inhibitor AZD1897 (Figure 4) and the AKT kinase inhibitor AZD5363 (capivasertib, Figure 4) showed synergistic cytotoxicity in AML associated with mTOR and MCL1 pathway repression [54]. It will be interesting to evaluate dual PIM/PI3K inhibitors, such as IBL-202 [55,56] (undisclosed structure) or IBL-302 [57] (Figure 4), in this setting (see also below). SEL24-B489 (MEN1793; Figure 4) is a dual-type I PIM/FLT3 inhibitor based on the benzoimidazole scaffold showing broad activity in AML cell lines and primary AML blasts and efficacy on AML xenografts [58]; SEL24-B489 entered Phase I/II clinical trials on AML in 2017 (NCT03008187). Imidazo-pyridazine SGI-1776 (Figure 4) has a similar selectivity profile and inhibits the growth of both chronic lymphocytic leukemia (CLL) [59] and AML [60] cell lines, but was discontinued after entering clinical trials, including on relapsed/refractory leukemias (NCT01239108), for dose-limiting cardiac toxicity.

Genetic alterations of FLT3 occur in about 30% of AML cases, more commonly as an internal tandem duplication (FLT3-ITD, 25% of new cases) or as mutations in the tyrosine kinase domain (FLT3-TKD, 7–10% of new cases) [9,61]. In particular, WHO identifies FLT3-ITD as affecting the clinical outcome of AML, conferring poor prognosis, and negatively impacting patient management. [62,63]. As in AML patients, redundant activation of multiple signal transduction pathways, such as PI3K/AKT, MAPK, and JAK/STAT, was observed and linked to poor prognosis early on, the targeting of the oncogenic signal at more than one level in FLT3-targeted therapies has been long recognized as a promising strategy for AML treatment [64]. Dual inhibition of FLT3 in AML has been recently and exhaustively reviewed ([65] and references within); we will focus here on compounds with specific dual kinase inhibitory profile which have undergone clinical investigations in AML.

**Figure 5:**
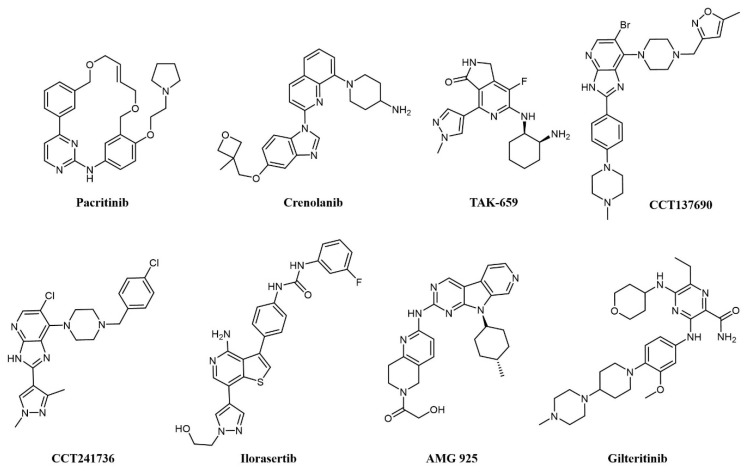
Chemical structure of multi-target fms-like tyrosine kinase 3 (FLT3) inhibitors used for AML treatment.

Of notice, dual FLT3/JAK2 inhibitor pacritinib (SB1518; Figure 5) [66] strongly inhibits FLT3 auto-phosphorylation and its downstream signaling pathways in AML cell lines and has efficacy in FLT3-ITD driven AML murine xenograft models [67]. Pacritinib has undergone clinical trials in combination for the treatment of AML in FLT-ITD-bearing (NCT02323607) and older patients (NCT02532010). Crenolanib (CP-868–596; Figure 5) is a type I inhibitor of PDGF with sub-nanomolar potency against FLT3 [68]. Crenolanib shows remarkable activity against resistance-conferring mutations of the kinase domain [69] and has entered a number of clinical trials against various indications, including two Phase II studies on relapsed/refractory AML (NCT01657682 and NCT03250338).

Spleen tyrosine kinase (SYK) was also recognized early as an important signaling partner of FLT3 [70], and overexpression of SYK confers resistance to selective FLT3 inhibitors [71]. Pyrido indolinone TAK-659 (Figure 5) was developed as an SYK inhibitor and showed strong activity against FLT3 and demonstrated efficacy against AML cell lines and in animal models [72]. TAK-659 has entered clinical trials on a number of indications, including a Phase I/II study as a single agent against relapsed or refractory AML (NCT02323113) to identify the maximum tolerated dose and evaluate preliminary efficacy on the disease.

Aurora kinases are a family of highly conserved serine-threonine protein kinases that have a key role in mitosis; overexpression of Aurora A has been consistently demonstrated in AML cell lines and patient cohorts [73]. Aurora kinase inhibitors were identified as promising agents in the treatment of FLT3-ITD-associated AML [73], providing a rationale for the development of dual FLT3-Aurora kinase inhibitors. The dual Aurora/FLT3 inhibitor CCT137690 (Figure 5) was identified following a medicinal chemistry program starting from an in-house Aurora inhibitor series [74] and was shown to inhibit the growth of FLT3 inhibitor-resistant AML cell lines [73]. CCT241736 (Figure 5) is a more advanced analog of CCT137690, with a favorable selectivity and PK profile and no hERG inhibition [74]. CCT241736 has significant in vivo efficacy against FLT3-ITD human tumor xenograft models and against FLT3-ITD cells with high in vitro relative resistance to the FLT3 inhibitors quizartinib and sorafenib. CCT241736 was also effective on primary samples from AML patients, including those with disease resistance to FLT3 inhibitor quizartinib [75]. Ilorasertib (ABT-348; Figure 5), an Aurora/VEGF inhibitor also potent against FLT3 [76], demonstrated clinical response in 3 out of 38 AML patients in a Phase 1 dose-escalation clinical study [77].

Cyclin-dependent kinase 4 (CDK4) is a downstream effector of growth factor activation, involved in the p16INK4a-CDK4-Rb axis in cancer development, which has a significant role in AML [78]. In preclinical studies, orally available FLT3/CDK4 dual kinase inhibitor AMG-925 (Figure 5) showed efficacy in AML tumor models and inhibited signaling in sorafenib-resistant AML cell lines [78].

In addition, c-Fes, a non-receptor tyrosine kinase involved in hematopoietic cells growth, survival, and differentiation as well as innate immune responses, is implicated in AML as a signaling partner of FLT3 [79]. In a recent work, a series of c-Fes inhibitors with anti-FLT3 activities in the nanomolar range have been shown to cause growth arrest and induce apoptosis in FLT3-ITD+ AML cells, and specific activity to FLT3-resistant cell lines [80].

Another RTK associated with poor prognosis and resistance to therapy in AML is AXL [81,82], a member of the Tyro3-Axl-Mer (TAM) family involved in a number of other cancers [83]. Gilteritinib (ASP2215; Figure 5) is a type I kinase inhibitor based on a pyrazine-carboxamide scaffold showing sub-nanomolar potency against FLT3 and AXL, activity against resistance-conferring mutations of FLT3 in cells, and a favorable pharmacokinetic profile granting activity on AML xenograft models [84]. Gilteritinib entered various clinical trials on AML, including the Phase III multicenter ADMIRAL trial (NCT02421939) comparing gilteritinib to chemotherapy in relapsed/refractory AML with FLT3-mutations [85]. Excitingly, gilteritinib significantly extended the overall survival of the treated patients and showed a higher percentage of patients with remission compared to salvage chemotherapy while showing less adverse events [86]. On the grounds of these results, gilteritinib was approved by the US Food and Drug Administration (FDA) and by the European Commission for the treatment of relapsed or refractory AML.

### 1.2. Dual Kinase Targeting in CML

Chronic myelogenous leukemia (CML) is a hematopoietic stem cell disease characterized by the Philadelphia (Ph) chromosome, a shortened chromosome 22 that results from the translocation between chromosomes 9 and 22, that is present in over 90% of CML patients [93]. The fusion gene *BCR-ABL* (breakpoint cluster region-Abelson leukemia virus) resulting from this translocation encodes the BCR-ABL fusion tyrosine kinase, which causes cell cycle deregulation, apoptosis, and affects DNA repair and differentiation [94,95]. The development of tyrosine kinase inhibitors changed the therapeutic options for CML patients dramatically, improving the 10-year survival rate from approximately 20% to 80–90% [96]. The BCR-ABL inhibitor imatinib was the first targeted therapy approved for the treatment of CML, and the first protein kinase inhibitor approved as a cancer treatment [1,97]. Imatinib quickly became the therapeutic standard for the treatment of CML, owing to the fact that frontline therapy was found to induce durable responses in a high proportion of patients [98]; despite these impressive results, resistance to imatinib treatment emerged as a clinical problem, with a fraction of patients failing to achieve complete hematological response by 3 months (10% of patients) or complete cytogenic response (25% of patients) by 18 months after therapy start [98,99], and a higher rate of resistance among patients with advanced phase CML [100].

Various mechanisms of resistance to tyrosine kinase inhibitor (TKI) treatment in CML have been reported, mainly caused by point mutations of the kinase domain [101], target gene amplification [102], and activation of alternative signaling pathways [103]. Among the latter, the most characterized cooperating pathway involves the avian sarcoma viral oncogene homolog (SRC) Family Kinases (SFKs), whose activation has been shown to induce a BCR-ABL independent mechanism of imatinib resistance [104,105]; furthermore, phosphorylation (activation) of BCR-ABL by SFKs is required for full oncogenic activity [106]. This provides a strong rationale for the use of dual SFK/ABL inhibitors in Ph+ CML.

**Figure 6:**
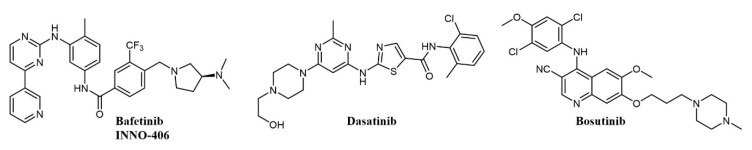
Chemical structure of dual SRC/ABL inhibitors used for chronic myelogenous leukemia (CML) treatment.

There are eight structurally related SFKs; the family is involved in RTKs, integrin, GPCRs, and immunoreceptor signaling [107]. Interestingly, the domain organization of ABL and SRC has significant homology [108], making possible the development of dual ATP-competitive SRC-ABL inhibitors. There are now five commercially available tyrosine kinase inhibitors for the treatment of Ph+ CML: imatinib, dasatinib, nilotinib, bosutinib, and ponatinib; of these, dasatinib and bosutinib (Figure 6) are dual SRC-ABL inhibitors [96]. Other advanced dual SRC-ABL inhibitors include FB2, a N-(thiazol-2-yl)pyrimidin-4-amine derivative (structure not completely disclosed) which shows in vitro and in vivo activity against TKI-resistant CML cell lines [109,110], and bafetinib (INNO-406, NS-187; Figure 6), an orally available inhibitor with activity on a number of ABL mutations which also selectively inhibits Lyn over other SRC family members and is able to penetrate the central nervous system (CNS) in murine models [111,112]. In a Phase I clinical trial on CML patients resistant or intolerant to imatinib and second-generation inhibitors, bafetinib achieved a 19% cytogenetic response rate [113]. Dasatinib (BMS-354825; Figure 6) was the first dual SRC-ABL inhibitor to enter the clinic and was developed starting from a series of substituted thiazole-5-carboxamides with activities against SRC and ABL and antiproliferative activity in CML cell lines and xenograft models [114]; besides SRC and ABL, dasatinib binds over 30 kinases, including major regulators of the immune system [115]. Dasatinib was initially approved in 2006 for the treatment of CML and Philadelphia-positive acute lymphoblastic leukemia (Ph+ ALL) patients resistant to therapy, including imatinib [116]; when compared with imatinib in a Phase III clinical trial at a dose of 100 mg/day, it showed higher molecular response rates [117]. Dasatinib has been the object of more than 300 clinical trials on CML and a number of other pathologies [118]. More recent clinical trials have shown encouraging efficacy of dasatinib at a lower dose, suggesting that future CML treatment could have a better safety profile and lower cost of care [119].

Bosutinib (SKI-606, Bosulif; Figure 6), also a dual SRC-ABL inhibitor, binds over 45 kinases with a selectivity profile different from dasatinib [120]. Bosutinib was developed from a series of 4-phenylamino-3-quinolinecarbonitriles SRC inhibitors with activity in vivo [121]. Bosutinib is able to overcome the majority of BCR-ABL mutations conferring resistance to imatinib, with the exception of T315I and V299L [122]. In clinical trials on the treatment of chronic phase CML, bosutinib has shown durable efficacy and manageable toxicity in patients resistant or intolerant to imatinib [123] and achieved higher and faster molecular response than imatinib as a first-line treatment [124]. Accordingly, bosutinib was first approved in 2012 for the treatment of resistant CML [125] and in 2017 for first-line treatment of chronic phase CML [96,126].

### 1.3. Dual Kinase Targeting in Lymphoid Tumors

The most recent WHO classification of tumors of hematopoietic and lymphoid tissues (WHO Classification of Tumors, Revised 4th Edition, 2017) reports six classes of lymphoid neoplasms of B-, T-, NK- and dendritic cell origin. Among them, several kinase-addicted diseases can be recognized. Ph+ ALL is a primarily pediatric cancer characterized by the presence of the t(9;22) (q34.1; q11.2) translocation and expression of the fusion oncogene BCR-ABL, akin to Ph+ CML. Hence, similar to CML, the use of imatinib brought great clinical improvement to patients [127]. Unfortunately, in contrast to CML, relapses are frequent due to the development of resistant disease, highlighting the need to develop new multi-targeted agents. A summary of the dual kinase inhibitors proposed for the treatment of lymphoid tumors is presented in Table 2 at the end of this section.

Several studies identified SFKs as key players in the development of Ph+ ALL, where they cooperate with the BCR-ABL fusion kinase to induce transformation [128]. Hence, co-targeting of BCR-ABL and SFKs by the dual ABL/SRC inhibitor dasatinib (Figure 6) allowed long-term survival of mice with Ph+ B-ALL [129]. This aminothiazole compound binds to the active conformations of ABL and SFKs with similar potency and induced complete remission of leukemia in imatinib-resistant patients [130]. Besides Ph+ ALL, other lymphoblastic leukemias can respond to ABL inhibitors, such as T-ALL expressing the NUP214-ABL1 fusion, but again, relapses are frequent. A relevant role of the SRC family kinases, in particular the Leukocyte C-Terminal Src Kinase (LCK), was identified in this disease as well [131]. The involvement of LCK suggested the use of dual ABL/SRC inhibitors. Indeed, NUP214-ABL1-positive cells and xenografts were exquisitely sensitive to dasatinib, and a patient with NUP214-ABL1 fusion was reported to obtain a complete remission with dasatinib [132].

**Figure 7:**
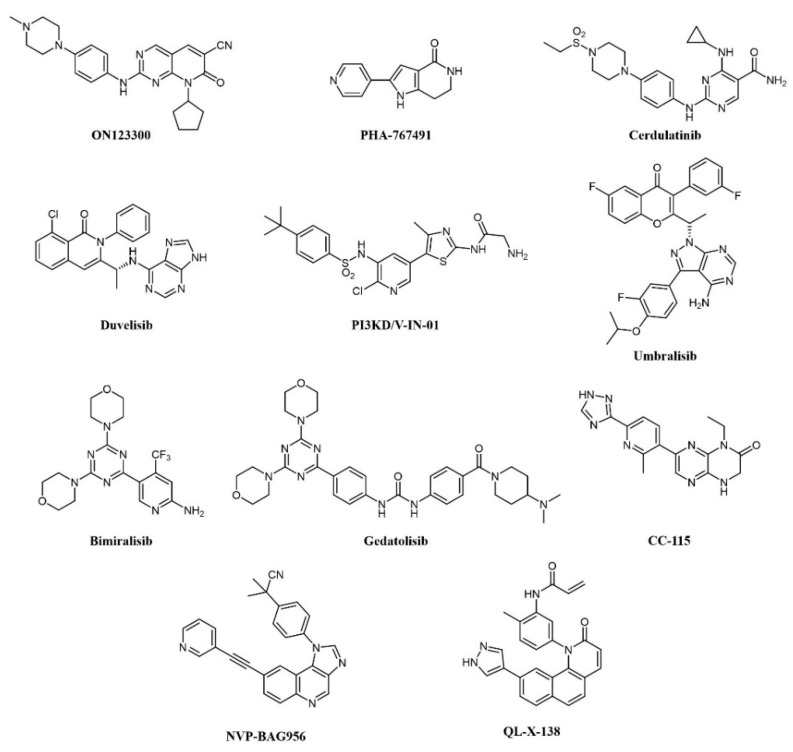
Chemical structure of dual kinase inhibitors used for lymphoid tumors.

Overexpression of cyclin D1 and consequent activation of CDK4/6 is a hallmark of mantle cell lymphoma (MCL); the CDK4 inhibitor palbociclib has shown modest clinical activity but was found to sensitize MCL cells to PI3K inhibitors [133]. Therefore, concomitant inhibition of CDKs and PI3K was pursued: ON123300 (Figure 7) is a novel pyrido-pyrimidine compound that inhibits CDKs 4 and 6, as well as ARK5 and PI3K-δ. Exploiting dual inhibition of CDK4/Rb and PI3K/AKT/mTOR circuits, the compound showed potent antitumor activity in vitro and in vivo [133]. Taking a similar approach, Natoni and colleagues aimed to simultaneously block two cell cycle regulators, CDC7 and CDK9, in CLL cells [134]. The authors used a pyrrolo-pyrimidinone compound, PHA-767491 (Figure 7), that is able to prevent cell division by blocking CDC7-induced activation of DNA replication origins [135]. PHA-767491 inhibited CLL cell proliferation induced by IL-4, a common mechanism of drug resistance in these cells. Surprisingly, the inhibitor also induced apoptosis in quiescent CLL cells from patients, probably through inhibition of CDK9-dependent transcription of MCL1. Again, this work highlights the value of dual targeting. The same compound was active in multiple myeloma cells [136], as well as in myeloid and solid tumors [137,138]. However, caution must be used because PHA-767491 also suppresses activation of normal T lymphocytes, potentially impairing antitumor immunity [139].

More recently, the precise elucidation of other pathways involved in cell transformation led to the design of a dual SYK/JAK kinase inhibitor for the treatment of lymphocytic leukemias and lymphomas. In these diseases, constitutive activation of SYK and JAK kinases has been observed [140,141]. In CLL and B-cell non-Hodgkin’s lymphoma (NHL), SYK is involved both in Bruton’s Tyrosine Kinase (BTK) pathway activation and IL4-driven resistance to BTK inhibitors, while JAK kinases transduce cytokine signaling, which supports tumor growth. A combination of SYK and JAK inhibitors synergistically inhibits cell proliferation, better than any single inhibitor. Hence, a dual SYK/JAK phenylamino-pyrimidine inhibitor, cerdulatinib (PRT062070; Figure 7), induced apoptosis in cancer cells but not in normal B cells, prevented drug resistance, and caused tumor inhibition in mice [142,143,144]. Cerdulatinib inhibits all JAK family kinases and is also efficacious in models of autoimmunity. The compound is currently under clinical investigation for relapsed/refractory CLL and other B-cell malignancies: In a Phase I trial, complete SYK and JAK pathway inhibition was achieved in whole blood of patients at tolerated exposures after oral administration. Objective tumor responses were observed in CLL and follicular lymphoma (FL) patients, including two complete responses. Three of eight CLL patients (38%) achieved a durable partial response lasting >200 days [145,146].

The involvement of class I PI3Ks in the onset of B-cell lymphoma is well documented [147]. First-generation pan-PI3K inhibitors have shown limited clinical efficacy in hematological cancers, probably due to unspecific inhibition of all isoforms and ensuing toxicity. Recently, isoform-selective inhibitors have shown better therapeutic efficacy. Duvelisib (IPI-145, INK-1197; Figure 7) is a dual PI3Kδ/γ quinoline inhibitor that shows selectivity over other class I PI3Ks [148,149]. In a Phase I trial in CLL patients, 56% and 83% responses were observed in chemotherapy-resistant and treatment-naïve patients, respectively. Similar results were obtained in a Phase II study in FL patients and a larger Phase III trial in patients with advanced hematological diseases, including NHL. The compound has now been approved by the FDA for the treatment of CLL and FL patients relapsed or refractory to chemotherapy [150,151]. PI3KD/V-IN-01 is a compound that simultaneously and selectively inhibits PI3Kδ and PIK3C3/Vps34, a class III PI3K isoform that can induce cytoprotective autophagy [152]. The compound showed superior antiproliferative activity against AML, CLL, and Burkitt’s lymphoma primary cells, compared to a PI3Kδ-only inhibitor (idelalisib) and inhibited tumor growth in vivo. Another PI3Kδ inhibitor, umbralisib (TGR-1202; Figure 7), also blocks casein kinase-1ε (CK1ε). Targeting of CK1ε has been shown to cooperate with idelalisib in hematological malignancies and to alleviate some immune-mediated adverse effects observed with pan-PI3K inhibitors. Thus, umbralisib was shown to possess comparable antitumor activity but lower toxicity in murine models of CLL [153]. The drug is being evaluated in CLL and B-cell NHL patients with encouraging results [154,155].

A number of PI3K inhibitors are described as dual PI3K/mTOR inhibitors. As discussed above, these compounds are likely to achieve a deeper suppression of the entire PI3K/AKT/mTOR pathway, compared to PI3K-specific inhibitors and, indeed, dual PI3K/mTOR inhibition is an established strategy for the treatment of AML. There is also strong evidence that simultaneous PI3K/mTOR inhibition induces better preclinical antitumor activity than single targeting in lymphoma [156]. Several PI3K/mTOR dual inhibitors have been developed in recent years, also thanks to a good structural similarity between the two kinases [157]. One such compound, bimiralisib (PQR309; Figure 7), was shown to inhibit all class I PI3K catalytic enzymes and mTOR with low nanomolar potency selectively while sparing the rest of the kinome [158]. Bimiralisib showed antitumor activity in preclinical lymphoma models [156] and is currently in clinical development for lymphoid malignancies. In a Phase I/II trial, clinical benefit was obtained in 8/11 lymphoma patients, including one complete remission (CR) [159]. Expanded cohorts are currently being enrolled in the trial. Gedatolisib (WYE-129587/PKI-587/PF-05212384; Figure 7), a PI3Kα/mTOR inhibitor, has been investigated in preclinical leukemia models, including T-ALL and Ph-like ALL, with very good results [160,161]. Dactolisib (see also above; Figure 3) inhibits cell growth and induces apoptosis in ALL and follicular lymphoma [88,89,90,91,92]. However, clinical investigations have mainly focused on solid tumors. Compound PI-103 (Figure 3) was tested on a panel of T-ALL cell lines and primary patients’ cells with constitutive activation of PI3K/Akt/mTOR pathway, showing potent cytotoxicity accompanied by complete inactivation of AKT, p70S6K, ribosomal S6 protein, and 4E-BP1 [87]. The importance of the mTOR pathway in cancer development further supported other bi-specific drug development programs, such as the dual mTOR/DNA-PK inhibitor, CC-115, or the PI3K/PDK1 inhibitor, NVP-BAG956 (Figure 7). DNA-PK is a Ser/Thr kinase that phosphorylates AKT in response to DNA damage, and, therefore, its inhibition can enhance the efficacy of anticancer treatments, and, in particular, of AKT/mTOR inhibitors. Indeed, CC-115 showed good preclinical and clinical activity in CLL [162,163]. NVP-BAG956, acting at two levels along the PI3K/mTOR pathway, showed potent cytotoxic effects against T-ALL cell lines and primary patients’ samples, outperforming drugs hitting a single target, such as PI3K, mTOR, or AKT inhibitors, which achieved comparable effects when combined, however [164].

In B-cell neoplasms, the roles of BTK and PI3K have been well established. Indeed, inhibitors of these kinases, such as ibrutinib and idelalisib, have shown efficacy against these cancers [166]. Liu et al. from the ShanghaiTech University (China) designed a series of dual BTK/PI3Kdelta pyridinone inhibitors, which simultaneously block BTK and PI3K pathways and inhibit Burkitt’s lymphoma cells growth [167,168]. In CLL cells, the dual PI3K/PIM kinase inhibitor IBL-202 suppressed cell proliferation and migration in hypoxic conditions where idelalisib failed [56]; the compound also showed efficacy in multiple myeloma [55]. Along similar lines of research, a BTK/MNK (Mitogen-Activated Protein Kinase Interacting Kinase) dual inhibitor was developed for lymphoma and leukemia (QL-X-138; Figure 7), which interestingly establishes covalent binding to BTK and a standard reversible binding to MNK [165]. MNK kinases are downstream effectors of various intracellular pathways and can induce transformation: their inhibition synergizes with upstream kinases blockade. QL-X-138 showed superior activity compared to a BTK inhibitor.

### 1.4. Other Dual Activity Inhibitors in Leukemia

**Figure 8:**
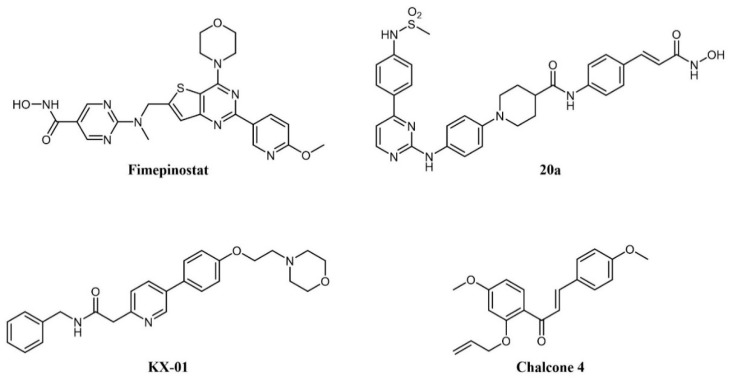
Chemical structure of dual-target kinase inhibitors.

As cancer is generally a multifactorial disease, effective therapeutic approaches make use of a combination of drugs endowed with different mechanisms of action (e.g., a kinase inhibitor in association with an antimetabolite [169]). While dual kinase inhibitors hit different targets belonging to the same protein family, a new concept of “dual-targeting” has been developed in recent years that makes use of a single drug endowed with more than one mechanism of action. Such compounds are obtained by merging the pharmacophore moieties of two different drugs into a single compound. Besides blocking different pathways at the same time, these compounds also have the advantage of single and more predictable pharmacokinetics. As an example, several reports have shown the synergistic potential of kinase and HDAC inhibitor combinations in cancer [170,171,172]. Hence, merging the two activities in one molecule would be a further step to increasing therapeutic activity. A medicinal chemistry effort aimed at incorporating HDAC inhibitory function into a PI3K inhibitor pharmacophore led to the identification of fimepinostat (CUDC-907; Figure 8), a PI3K/HDAC dual inhibitor for the treatment of lymphoid cancers [173,174,175]. In a Phase I trial, the drug achieved an objective response in 5/9 patients with relapsed or refractory Diffure Large B-Cell Lymphoma (DLBCL) [176]. While its safety profile was consistent with those of FDA-approved HDAC inhibitors and PI3K inhibitors, fimepinostat showed superior tolerability as it did not lead to the severe side effects associated with HDAC and PI3K inhibitors (e.g., hepato- and cardiotoxicities) [176]. Notably, in animal models, the co-administration of a PI3K inhibitor and an HDAC inhibitor was not tolerated, whereas the use of fimepinostat did not show signs of appreciable toxicity at therapeutic doses [177]. Preclinical studies also suggested a potential application in the treatment of CLL [178], mantle cell lymphoma (MCL) (174), and AML [179]. The compound is currently undergoing Phase 1 clinical investigation in children and young adults with relapsed or refractory solid tumors, CNS tumors, or lymphoma (ClinicalTrials.gov Identifier: NCT02909777).

Several rationally designed JAK2/HDAC6 inhibitors have also been reported [180]. The most promising compound in the series (20a in the paper; Figure 8) showed comparable potency against the targets (IC_50_ JAK2 = 8 nM; IC_50_ HDAC6 = 46 nM), which prolonged the survival of mice in an AML xenograft model, and counteracted spleen enlargement. A preliminary evaluation of the pharmacokinetic profile was also performed. 

Tirbanibulin (KX-01; Figure 8) is a novel compound equipped with tyrosine kinase inhibitory as well as antimicrotubule activity. It is, in fact, a peptidomimetic compound that inhibits at the same time SRC kinase (through interference with substrate binding) and microtubule polymerization, thus providing a comprehensive suppression of the cell migration machinery [181]. The compound is under clinical investigation for several indications, including lymphoma (ClinicalTrials.gov Identifier: NCT00646139). A recent report identified natural chalcone compounds as dual FLT3 and microtubule polymerization inhibitors [182]. The authors then derived synthetic analogs, the most active of which (chalcone 4; Figure 8) simultaneously reversed activation of drug-resistant FLT3-ITD^D835Y^ mutant and tubulin polymerization in vitro. 

### 1.5. PROTACs

**Figure 9:**
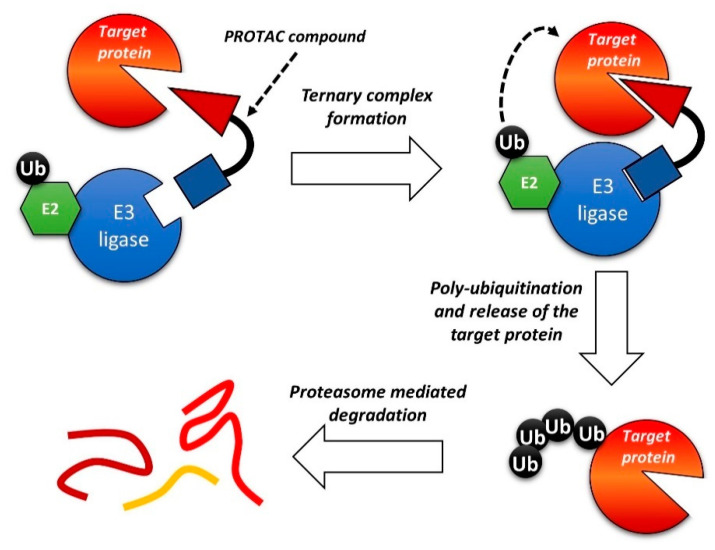
Schematic representation of the Proteolysis Targeting Chimeras (PROTAC) strategy.

One of the most interesting recent developments in this field relates to Proteolysis Targeting Chimeras (PROTACs) compounds. It is a new class of bifunctional molecules, composed of 1) a ligand that binds a biomolecular target; 2) a ligand that recognizes the E3 ubiquitin ligase; 3) a linker between 1 and 2 [183,184,185,186,187]. The interaction with E3 ubiquitin ligase promotes the ubiquitination of the biomolecular target and, consequently, the degradation of the protein by the proteasome. By this mechanism, PROTACs can reduce the intracellular concentration of a given protein, leading to a prolonged and marked inhibitory effect. Despite the very recent development of such a strategy, several papers and reviews on the PROTACs strategy can be found in the literature [188,189,190,191]. A general scheme of the PROTACs strategy is reported in Figure 9.

**Figure 10:**
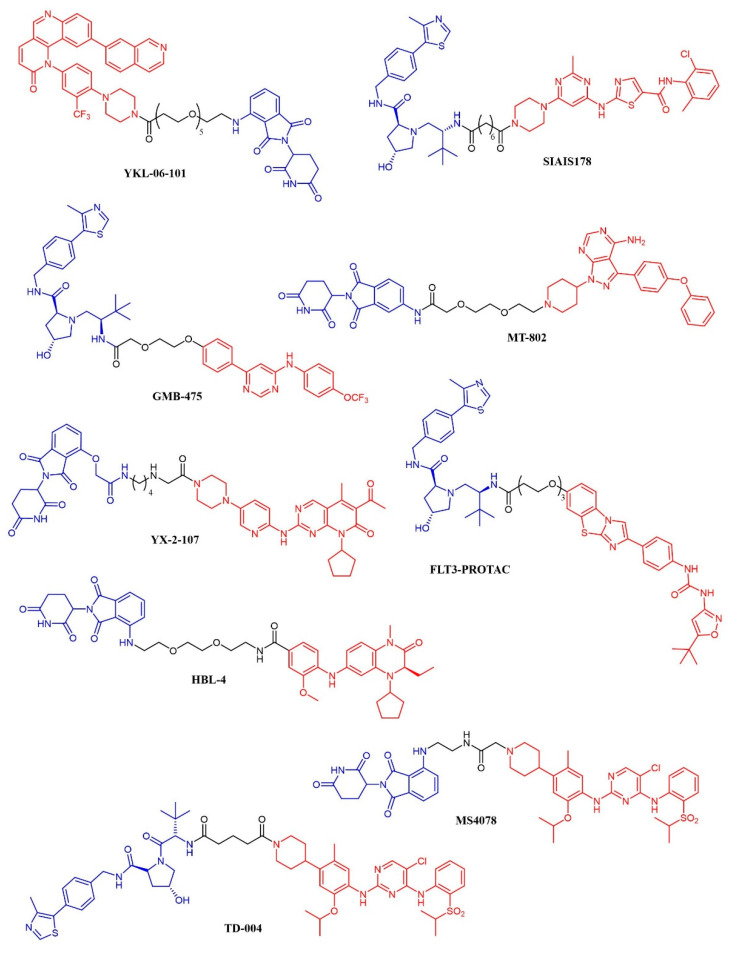
Chemical structure of kinase inhibitor derived PROTACS. Ubiquitine ligase recognizing moiety is depicted in blue; kinase recognizing moiety is depicted in red; linker is depicted in black.

The PROTAC approach was recently applied to BCR-ABL-driven leukemia. Various reports identified cyclin-dependent kinase 8 (CDK8) as a key mediator of Ph+ B-ALL, also through kinase-independent functions. In this context, CDK8 was shown to activate the PI3K/mTOR pathway. Thus, combined inhibition of CDK8 and mTOR was pursued: The authors showed the efficacy of a dual CDK8/mTOR inhibitor, YKL-06-101 (Figure 10; Table 3), which is also a CDK8 degrader [192]. Degradation of CDK8 was necessary because the simple biochemical inhibition was insufficient to cause cell death due to the kinase-independent activity of CDK8. The compound was derived from the optimization of Torin1, a potent mTOR inhibitor [193], towards dual mTOR/CDK8 inhibition; this fragment was then linked to thalidomide to add the degrader function. YKL-06-101 was equally active in Ph+ and Ph- B-ALL cells. Interestingly, this compound recapitulates both the dual kinase and the PROTAC approach.

A series of dasatinib-based PROTACs has been recently reported (194). The compounds differed from the linker length and were preliminarily screened against K562 cells. The most promising compound (SIAIS178; Figure 10) was found to induce the selective degradation of BCR/ABL kinase and several mutants thereof. Notably, the modification of the dasatinib structure caused a narrower selectivity profile of the PROTAC compound. When tested in a murine xenograft model of K562 cells, the compound effectively reduced the tumor progression but did not outperform dasatinib. The GNF-5 allosteric modulator of BCR-ABL kinase has been modified according to the PROTAC strategy, leading to GMB-475 (Figure 10). The compound induced a time- and dose-dependent degradation of the target protein. The potential antineoplastic activity was assessed in K562 and Ba/F3 cell lines and primary CML patient samples. GMB-475 induced the degradation of the target protein in CML stem cell populations also, but without causing marked apoptosis, further confirming that CML stem cells’ survival is not dependent on BCR-ABL (195).

MT-802 (Figure 10) is an ibrutinib-based PROTAC active against primary CLL patient samples. The compound outperformed the parent kinase inhibitor, resulting in the ability to induce the degradation of both wt-BTK and the ibrutinib-resistant mutant BTK^C481S^ {196}. No in vivo experiments have been reported so far.

YX-2-107 (Figure 10) is a palbociclib-derived PROTAC that induces the degradation of CDK6 in Ph+ ALL cells [197]. When tested in vivo in Ph+ ALL xenograft models, the compound suppressed the number of cells in the S phase with the same potency as palbociclib. However, the PROTAC derivative abrogated the expression of CDK6 (that is, instead, upregulated upon treatment with palbociclib) and, at a lower extent, of CDK4. The pharmacokinetic properties of YX-2-107 were not optimal, and the compound needs further improvement.

The enhancement in the selectivity of PROTAC derivative of kinase inhibitor was also observed for a quizartinib-based PROTAC, that resulted active both in vitro and in vivo against AML models [198]. The compound (named FLT-PROTAC; Figure 10) was able to abrogate the FLT3-ITD expression and showed higher efficacy than quizartinib in vitro and an MV4-11 xenograft model. 

HBL-4 (Figure 10) is a dual BRD4/PLK1 degrader, obtained through the modification of the dual inhibitor BI2536 according to the PROTAC strategy [199]. HBL-4 effectively reduced the tumor growth in an MV4-11 xenograft AML model and outperformed the parent compound both in vitro and in vivo.

Finally, the same approach was recently developed in the context of ALK-positive lymphoma: three groups described degraders of ALK kinase based on phenylamino-pyrimidine ALK inhibitors (NVP-TAE684 or ceritinib) and either pomalidomide (compounds **9**, **10**, **11**, **12** [202]; compounds MS4077 and MS4078 [200]) or a different von Hippel-Lindau ligand (TD-004 [201]). The latter was shown to have in vivo efficacy in a xenograft model.

## 2. Conclusions

There has been great excitement for rationale targeting during the past 20 years in hematologic cancer therapy: several breakthroughs have been possible via the precise understanding of tumor biology. On this basis, the quest for super-selective drugs yielded several candidates for next-generation drugs. Unfortunately, genetic heterogeneity of tumors, and intricate cross-talks of different oncogenic pathways, bring complex evolutionary trajectories and inevitable resistance to monotherapies. However, we must not be discouraged by this complexity. Drug combinations hold promise to hit multiple drivers of tumorigenesis, but the pharmacokinetics (and safety) of multi-drug treatments may pose some difficulties. In contrast, rational dual inhibitors merge two important features of a magic bullet: dual targeting and simple metabolism and toxicology profiles, as witnessed by the clinical success and relatively speedy development of agents, such as gilteritinib for AML, dasatinib, bosutinib, and bafetinib for CML and duvelisib for lymphoid tumors. The expanding field of dual targeting will increase our chances of achieving a safe and clinically manageable poly-pharmacology approach. 

## Figures and Tables

**Table 1:** Molecular targets and clinical studies of dual agents proposed for the treatment of acute myeloid leukemia (AML).

Compound	Targets	Disease	Status	Clinical Trials	Ref.
**OSI-027**	mTORC1 mTORC2	AML	Phase 1	NCT00698243 (5% SD)	[31,32,33]
**AZD8055**	mTORC1 mTORC2	Solid and hematologic	Discontinued	NCT00731263 (18% SD)	[34,35]
**AZD2014/Vistusertib**	mTORC1 mTORC2	DLBCL	Phase 2	NCT02752204	[38,39]
**TAK-228/MLN0128**	mTORC1 mTORC2	Lymphoma	Phase 1/2	NCT02727777	[42,43]
**PP242**	mTORC1 mTORC2	AML	Preclinical	-	[40]
**PI-103**	PI3K mTOR	AML, ALL	Preclinical	-	[47,87]
**Dactolisib**	PI3K mTOR	AML, ALL, FL	Phase 1	NCT01756118 (30% ORR)	[50,51,52,88,89,90,91,92]
**NVP-BGT226**	PI3K mTOR	AML	Preclinical	-	[51]
**SEL24-B489**	PIM1 FLT3	AML	Phase 1/2	NCT03008187	[58]
**SGI-1776**	PIM1 FLT3	AML	Discontinued	NCT01239108	[60]
**Pacritinib**	FLT3 JAK2	AML	Phase 2	NCT02323607 (23–50% CR) NCT02532010	[66,67]
**Crenolanib**	PDGF FLT3	AML	Phase 3	NCT01657682 NCT03250338	[68,69]
**TAK-659**	SYK FLT3	AML	Phase 1/2	NCT02323113	[72]
**CCT137690**	Aurora FLT3	AML	Preclinical	-	[73,74]
**CCT241736**	Aurora FLT3	AML	Preclinical	-	[74,75]
**Ilorasertib**	Aurora VEGFR FLT3	AML	Phase 1	NCT01110473 (8% ORR)	[76,77]
**AMG-925**	FLT3 CDK4	AML	Preclinical	-	[78]
**Gilteritinib**	FLT3 AXL	AML	Approved	NCT02421939 (28% CR)	[84,86]

**Table 2:** Molecular targets and clinical studies of dual agents proposed for the treatment of lymphoid tumors.

Compound	Targets	Disease	Status	Clinical Trials	Refs
**ON123300**	PI3K CDK4/6	MCL	Preclinical	-	[133]
**PHA-767491**	CDC7 CDK9	CLL, MM	Preclinical	-	[134,135,136,137,138,139]
**Cerdulatinib**	SYK JAK	CLL, FL	Phase 1	NCT01994382 (30–38% ORR)	[142,143,144,145,146]
**Duvelisib**	PI3K-δ PI3K-γ	CLL, NHL, FL	Approved	NCT01882803 (47% ORR)	[148,149,150,151]
				NCT02004522 (74% ORR)	
				NCT01476657 (56–83% ORR)	
**PI3KD/V-IN-01**	PI3K-δ PIK3C3	AML, CLL, Burkitt’s lymphoma	Preclinical	-	[152]
**Umbralisib**	PI3K-δ CK1ε	CLL, NHL	Phase 1	NCT01767766 (37% ORR)	[153,154,155]
**Bimiralisib**	PI3K mTOR	Lymphoma	Phase 1/2	NCT03127020 (73% ORR)	[158,159]
**Gedatolisib**	PI3K-α mTOR	ALL	Preclinical	-	[160,161]
**CC-115**	mTOR DNA-PK	CLL	Phase 1	NCT01353625 38% PR, 25% SD	[162,163]
**NVP-BAG956**	PI3K PDK1	ALL	Preclinical	-	[164]
**QL-X-138**	BTK MNK	B-cell lymphoma	Preclinical	-	[165]

**Table 3:** Molecular targets and target disease of Proteolysis Targeting Chimeras (PROTAC) agents.

Compound	Targets	Disease	Refs
YKL-06-101	CDK8 mTOR	ALL	[192]
SIAIS178	BCR/ABL	CML	[194]
GMB-475	BCR/ABL	CML	[195]
MT-802	BTK	CLL	[196]
YX-2-107	CDK6	ALL	[197]
FLT-PROTAC	FLT3	AML	[198]
HBL-4	BRD4 PLK1PLK1	AML	[199]
MS4078	ALK	ALCL	[200]
TD-004	ALK	ALCL	[201]
